# Selection for tandem stop codons in ciliate species with reassigned stop codons

**DOI:** 10.1371/journal.pone.0225804

**Published:** 2019-11-26

**Authors:** Ira Fleming, Andre R. O. Cavalcanti

**Affiliations:** Department of Molecular Biology, Pomona College, Claremont, CA, United States of America; Texas A&M Health Science Center, UNITED STATES

## Abstract

The failure of mRNA translation machinery to recognize a stop codon as a termination signal and subsequent translation of the 3’ untranslated region (UTR) is referred to as stop codon readthrough, the frequency of which is related to the length, composition, and structure of mRNA sequences downstream of end-of-gene stop codons. Secondary in-frame stop codons within a few positions downstream of the primary stop codons, so-called tandem stop codons (TSCs), serve as backup termination signals, which limit the effects of readthrough: polypeptide product degradation, mislocalization, and aggregation. In this study, ciliate species with UAA and UAG stop codons reassigned to code for glutamine are found to possess statistical excesses of TSCs at the beginning of their 3’ UTRs. The overrepresentation of TSCs in these species is greater than that observed in standard code organisms. Though the overall numbers of TSCs are lower in most species with alternative stop codons because they use fewer than three unique stop codons, the relatively great overrepresentation of TSCs in alternative-code ciliate species suggests that there exist stronger selective pressures to maintain TSCs in these organisms compared to standard code organisms.

## Introduction

In the standard model of protein translation in eukaryotes, the stop codons UAA, UAG, and UGA are not recognized by tRNAs bearing amino acids and instead pair with eukaryotic release factors (eRFs), which trigger the hydrolysis of the ester bond linking the nascent polypeptide to the ribosome-mRNA complex, terminating translation [1]. Stop codon recognition, however, is subject to error. Stop codon readthrough, or just “readthrough,” refers to an instance in which the ribosome’s encounter with a stop codon does not trigger termination. The frequency of readthrough can be as low as 0.0001% or as high as 10% for a gene’s mRNA transcript depending on the organism, gene, and context of the stop codon, though global rates of readthrough are relatively low (e.g. ~0.3% of total transcripts in *Saccharomyces cerevisiae*) [2–5]. When readthrough occurs, part or all of the downstream 3’ UTR of the mRNA transcript is translated, resulting in the addition of extra amino acids onto the C-terminus of the polypeptide, a C-terminal extension [6]. Failure to terminate translation at the end of a gene may be the result of a mutated release factor or nonstop mutations that alter a stop codon to a sense codon, however, these mutations are relatively rare while readthrough itself is ubiquitous across the genome [7]. In these instances of mutation-free readthrough, a near-cognate tRNA recognizes a stop codon as sense and outcompetes eRF1, despite the imperfect pairing [8,9].

Though readthrough does have adaptive advantages in some conditions and some viral and even some eukaryotic genomes employ “programmed” readthrough of stop codons to produce fusion genes, there is generally a fitness cost to high rates of global readthrough [10–12]. There are two predominant and mutually exclusive pathways for a transcript undergoing readthrough, both of which bear their own costs for the cell. Either the ribosome reads the entire 3’ UTR and reaches the poly(A)-tail or the ribosome reads until a secondary in-frame stop codon which induces termination in the 3’ UTR [2]. In the first scenario, the ribosome stalls in the poly(A)-tail and the nonstop decay (NSD) pathway is initiated, in which the mRNA subject to readthrough is degraded by the exosome and the nascent polypeptide chain is ubiquitinated and degraded by the proteasome [13,14]. In the second scenario, a protein is produced with a C-terminal extension. A readthrough product such as this is generally ubiquitinated and degraded as well, with some longer extensions acting as degrons; in situations where a gene has high rates of readthrough, the longer the extension, the greater the proportion of readthrough products that are degraded [2,3,15]. A high enough rate of degradation resulting from constitutive readthrough may then lead to pathogenic effects by way of the loss of function for affected genes–at least twenty nonstop mutations related to human disorders were identified wherein the C-terminally-extended proteins were ubiquitinated and degraded by the proteasome system [15]. If the readthrough products persist, however, C-terminal extensions may cause mislocalization or aggregation of affected proteins, resulting in disorders like dementia, if the readthrough is constitutive [16,17].

The consequences of readthrough (NSD/degradation of polypeptide products and mRNAs, and misfolding, mislocalization, and aggregation of proteins) incentivize the evolution of cellular mechanisms that limit the length of C-terminal extensions during readthrough or prevent readthrough entirely. One safety mechanism which can mitigate the effects of readthrough is the inclusion of backup in-frame stop codons immediately or a few tri-nucleotide positions downstream of primary stop codons, which are considered to be “tandem stop codons” (TSCs) [18,19]. When readthrough occurs in TSC-bearing genes, only a limited number of amino acids -or none at all if the stop codons are adjacent—are added onto the C-terminus of the polypeptide before the ribosome encounters the TSC, which then may effectively terminate translation. A short or nonexistent C-terminal extension eliminates cases where mRNA and polypeptide breakdown as a result of NSD would take effect, reduces the degradation of fully formed readthrough products [2], and in theory, might prevent the misfolding that results in mislocalization/aggregation, thereby increasing the fitness of the cell.

TSCs were first recognized as adjacent stops in bacteriophages and in *Escherichia coli* [18,19], though other studies expanded the investigation of TSCs to eukaryotic organisms and non-adjacent codons [20, 21]. In yeast, researchers identified an excess of stop codons at the first three in-frame positions downstream of UAA primary stop codons in four species of *Saccharomyces* and suggested that selection for this feature is stronger in high-expression genes [20]. In plants, a weak bias for stop codons in the +1 position of the 3’ UTR was observed in *Arabidopsis thaliana* and O*ryza sativa* [21].

Ciliates are prime candidates for the investigation of TSCs because stop codon recognition is altered in several ciliate species by way of stop codon reassignment. In some ciliates, the UAA and UAG stop codons have been reassigned to code for glutamine (Gln), leaving UGA as the sole stop codon, while in others, UGA is reassigned to code for cysteine (Cys) or tryptophan (Trp) [22, 23]. In these cases of stop codon reassignment, two aspects of codon recognition are altered: (1) tRNAs must develop to read the former stop codon or a near-cognate tRNA must efficiently pair with the codon, and (2) eRF1 must lose its ability to recognize the codon as a termination signal or at least its ability to effectively outcompete tRNAs at that codon. These changes are intrinsically related to translation termination and might possibly lead to different patterns of TSC usage in these organisms.

A preliminary exploration of TSCs in ciliates reported higher than expected levels of TSCs in *Tetrahymena thermophila* and *Paramecium tetraurelia*, both of which have the UAR stop to Gln reassignment [24]. There was also an observed excess of TSCs in yeast, though it was not so great as that seen in the alternative code ciliates. The authors suggest that there may be stronger forces of selection for TSCs in the *T*. *thermophila* and *P*. *tetraurelia* genomes, which may be related to higher rates of readthrough or some other evolutionary driver. They also propose that ciliate release factors may include mutations related to altered specificity as well as termination efficiency, which could lead to a theoretical increased rate of readthrough [24].

Evidence for a relationship between alternative genetic codes and readthrough/TSCs continues to be uncovered in ciliates with ambiguous codes. Recently, the ciliate species *Condylostoma magnum* and *Parduzcia sp*., as well as the dinoflagellate *Amoebophrya sp*.*–*from the same superphylum of ciliates, alveolates–have been identified, none of which have dedicated stop codons [25, 26]. In *C*. *magnum*, for example, UAA and UAG code for Gln and UGA for tryptophan (Trp), though all three codons may also trigger termination. The reading of one of these ambiguous codons as sense is similar to a readthrough event: a codon which often functions as a stop fails to initiate termination and is treated as sense by a tRNA. *C*. *magnum*, upon further investigation, was found to possess no cognate tRNA match for UGA (only the near-cognate CCA-Trp tRNA), making the comparison to readthrough even more appropriate. One might predict that because *C*. *magnum* reads all its stop codons as sense in certain contexts, it would be especially permissive of readthrough, and would therefore have a high number of TSCs. The authors do note frequent TSCs that occur at an average of six codon positions downstream of primary stop codons that could function effectively as a backstop in the event of readthrough though they do not claim to see unusually high rates of readthrough in *C*. *magnum* [25]. Even if termination is highly efficient *in C*. *magnum*, however, other ciliates with complete reassignments that may have evolved from ambiguous codes could possess some lingering codon ambiguity tolerance, possibly permissivity to readthrough, and greater incentives to maintain TSCs in the genome.

Given the dearth of knowledge surrounding TSCs, a multi-species screen is due to determine strength of selection for TSCs in eukaryotic species with and without stop codon reassignments. The release of multiple new annotated ciliate genomes now presents an opportunity to investigate statistical excesses of TSCs in organisms with alternate codes. Relatively large excesses of TSCs observed in ciliate species with reassigned stop codons such as *T*. *thermophila* and *P*. *tetraurelia* [24], and the observed stop codon ambiguity in ciliate species like *C*. *magnum* [25], suggest that ciliate species with reassigned stop codons may have stronger forces of selection for TSCs than do standard code eukaryotic species.

## Methods

### Data sources

We used the genomes of nine ciliates that reassign UAR codons to Gln, one ciliate that reassigns the UGA codon to Cys, and one ciliate that uses the standard code. The most current annotated macronuclear genome sequence and general feature format (gff) genome annotation files for the ciliate species *Oxytricha trifallax*, *Ichthyophthirius mutifilis*, *Tetrahymena borealis*, *Tetrahymena ellioti*, *Tetrahymena malaccensis*, *T*. *thermophila*, *Stylonychia lemnae*, and *Stentor coeruleus*, were obtained from the ciliates.org database [27].The genome sequence and gff files for the ciliates *P*. *tetraurelia* and *Pseudocohnilembus persalinus* were downloaded from NCBI (accession numbers for all genomes obtained from NCBI are given in S1 Table). Finally, we obtained the genome sequence for *Euplotes octocarinatus* from the *Euplotes octocarinatus* genome database (EOGD) [28]. In the case of *E*. *octocarinatus*, a gff file with annotation was not available, and we relied instead on a file containing predicted gene sequences [28]. The genome sequence and gff files for all other organisms in this analysis were downloaded from NCBI.

In addition to ciliate genomes, we compiled the genomes of an additional 72 standard code eukaryotes. Because ciliates are part of the greater alveolate superphylum, we separated the standard code non-ciliate genomes in two groups: 12 non-ciliate alveolates (referred as “other alveolates”) and 60 phylogenetically diverse eukaryotes (composed of 10 stramenopiles, 1 haptophyte, 5 amoebozoa, 1 rhizaria, 5 euglenozoa, 2 parabasilids, 4 red algae, 10 viridiplantae, 10 fungi, 2 choanoflagellates and 10 metazoa).

The diplomonad *Spironucleus salmonicida* also reassigns UAA and UAG to Gln, so we treated the diplomonad group separately from the other eukaryotes. We obtained the genome of *S*. *salmonicida*, and of two other diplomonads that use the standard code from NCBI.

A list of all organisms used in the analysis with their phylogenetic classification and download location/accession number is given in S1 Table.

### Production of 3’ UTR stop codon overrepresentation scores

The start and stop codon coordinates of each gene within the genome assembly FASTA files were obtained from the corresponding gff files for each organism, with the exception of *E*. *octocarinatus* for which a gff file was not available. For this organism we downloaded the genome assembly and the gene prediction files and mapped the predicted genes to the genome sequence to determine the stop codon coordinate for each gene.

For each genome, the first 20 nucleotides following the stop codons of each gene with an annotated ATG start codon and a stop codon were extracted to capture the 3’ UTR of the resulting mRNA transcript. The reverse complement was produced for genes on the minus strand. Given the short 3’ UTRs of some ciliate species (the median UTR length for heterotrichs is 21-23nt [25]), we chose to use a small UTR region of 20 nucleotides past the annotated stop codon in this analysis and compiled those regions into a UTR library for each organism. Another justification for using such small UTRs is that the closer to the real stop codon a TSC is, the shorter the C-terminal extension and the less likely that the protein product will be degraded or misfold.

Given the rarity of readthrough and the efficacy of a single secondary stop codon, we analyzed only the first-occurrence tandem stop codons in each organism. A tandem stop codon was only included in the observed count if it was the first stop codon in each 3’ UTR and expected counts were calculated to accommodate for this condition as well. Note that, although these triplets are untranslated, we will refer to them as codons for convenience.

The number of observed first-occurrence stop codons at each 3’ UTR position is drawn from the UTR libraries for each genome. We count how many genes in the genome have a TSC in the first in-frame position downstream of the annotated codon (position +1 composed of the first three nucleotides past the primary stop codon). For the remaining genes, we count how many have a tandem stop at position +2 (nucleotides 4–6 following the annotated stop). We repeat this procedure up to position +6 (nucleotides 16–18 following the annotated stop). We represent the fraction of genes in the genome with first-occurrence tandem stops in each position by *obs*_*i*_.

To calculate the expected frequency of tandem stop codons in each in-frame position following the annotated stop, we determine the nucleotide composition of all the combined UTRs from an individual genome and use it to calculate an expected frequency for stop codons in each UTR position in the genome. The expected probability (*p*_*codon*_) of finding a given codon at any position in the 3’ UTR is:

$$p_{codon}=P(B_{1})\times P(B_{2})\times P(B_{3})$$ (1)

where P(B_N_) is the frequency of base B (which is at position N in the codon) in the compiled 3’ UTR library for the organism. For example, the *p*_*codon*_ for UGA in an organism which has an UTR composition of 30% A, 30% T, and 20% G is:

$$p_{UGA}=P(U)\times P(G)\times P(A)=P(0.3)\times P(0.2)\times P(0.3)=0.018$$

The *p*_*codon*_ for all stop codons used in the genome were added together to give the expected probability of a stop codon at any position within the UTR, *p*_*stop*._

*p*_*stop*_ gives the likelihood of codon occurrence, but not ‘first-occurrence’. At position *i* in a single UTR, where *i* starts at one and increases by one for each codon position past the annotated stop codon, the probability (*q*_*i*_) of finding a ‘first-occurrence’ stop codon at a given 3’ UTR position is:

$$q_{i}=p_{stop}\times {\left(1-p_{stop}\right)}^{i-1}$$ (2)

the (1−*p_stop_*)^*i*−1^ term represents the probability that all codons preceding position *i* were not stop codons. The expected first-occurrence stop codon counts for each in-frame 3’ UTR position (*exp*_*i*_) can then be calculated by multiplying *q*_*i*_ by the total number of 3’ UTRs in the dataset. *exp*_*i*_ is an expression of the null model in which the probability of a first-occurrence stop (*q*_*i*_) and the expected counts of first-occurrence stops (*exp*_*i*_) follow a negative binomial distribution where the stooping parameter is 1 and the probability of success is *p*_*stop*_. It is also assumed in this model that *p*_*stop*_ is independent and has the same distribution for all six positions in the 3’ UTR window.

A log ratio was then taken between the expected and observed counts to produce a stop codon overrepresentation score (SCO) for each position downstream of the primary stop codon:

$${SCO}_{i}=\log_{e}\left({obs}_{i}/{exp}_{i}\right)$$ (3)

The SCO for the +1 to +6 positions of the 3’ UTRs for each species was calculated. Scores above zero show overrepresentation of that codon at a given position, i.e., we observe a stop codon more frequently than expected by chance. In ciliates and in the diplomonad that reassign UAR to Gln, the combined SCO includes only the UGA codon; for standard code species the SCO was calculated as the log-ratio of the observed versus expected total combined counts of all three stop codons UAA, UAG and UGA; for *E*. *octocarinatus* the SCO was calculated using the stop codons UAG and UAA. SCOs are then combined into sets (ciliates, non-ciliate alveolates, and non-alveolate eukaryotes). The statistical significance of the differences between the SCOs in each of these organism groups was calculated using the non-parametric two-tailed Mann-Whitney test of significance.

The number of genes with at least one in-frame stop codon in the first six positions of their UTRs was also recorded for each organism. From that count and the total count of UTRs in the set, the percentage of genes with TSCs within six positions of the primary stop was calculated for each organism. Additionally, we calculated the expected number of genes with TSCs in the first six downstream codon positions by summing the *exp*_*i*_ for these positions. We can thus calculate a combined SCO for the first 6 codons positions as an overall stop codon overrepresentation score for the organism.

### TSC overrepresentation in specific genes

To determine if TSC tend to occur in highly expressed genes we further analyzed the genome of *T*. *thermophila* as this is the ciliate with the most available genetic data. We followed the procedure of [29], and used a codon usage bias measure, codon adaptation index, CAI [30], as a proxy for expression level. We used a set of 225 genes determined to be highly expressed in *T*. *thermophila* [31] as a reference to calculate the relative adaptiveness of each codon in this organism. We then calculated CAI for each *T*. *thermophila* gene, separated the genes in quartiles following their CAI values, and compared the percentage of genes with TSCs between groups.

A repository with the python scripts used in this study is available at GitHub: https://github.com/aroc110/FlemingCavalcanti2019.

## Results

### TSC analysis across species

**Fig 1:**
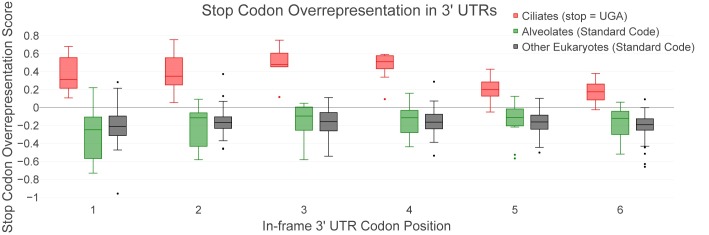
Box-plot comparing SCO scores in 3’ UTRs across species. The SCO scores for each in-frame position of their 3’ UTRs was calculated for each of 81 organisms. The scores are plotted at each of the first 6 in-frame positions of their 3’ UTRs and grouped in the following manner: 9 ciliates with the UAR stop codons reassigned to Gln (red), 12 standard code non-ciliate alveolates (green), and 60 standard code non-alveolate eukaryotes (black). Box plots are used to represent combined SCOs from all species in each grouping at each in-frame codon position in the 3’ UTR, with whiskers extending to a maximum of 1.5 interquartile range, the edges of the boxes at Q1 and Q3, and the central line indicating the median SCO at that position. Scores outside the whiskers’ range are shown as points.

Sets of 3’ UTRs were obtained for 86 different eukaryotic organisms (S1 Table), some with the UAR stop codon to Gln reassignment, one with a UGA to Cys reassignment, and the remainder with the standard set of stop codons. The log ratio of observed over expected counts of tandem stop codons at each in-frame 3’ UTR position gives a measure of relative representation where a score greater than zero shows overrepresentation of stop codons at that position. Box-plots of the SCOs for each 3’ UTR position for ciliates with the UAR stop to Gln reassignment, standard code non-ciliate alveolates, and standard code non-alveolate eukaryotes are given in Fig 1 (the values of the overrepresentation of stop codons in each downstream position for all species analyzed are given in S2 Table).

For ciliates with the UAR stop to Gln reassignment, the SCOs are mostly positive in the first six positions of their 3’ UTRs, with scores ranging from -0.049 to 0.756. Standard code non-ciliate alveolates and standard code non-alveolate eukaryotes have lower SCOs for these positions, ranging from -0.729 to 0.221 and from -0.958 to 0.373 respectively (Fig 1 and S2 Table). The SCOs are higher (p<0.001) for the ciliates than for both standard code non-ciliate alveolates and standard code non-alveolates at all the first six 3’ UTR codon positions as determined by a non-parametric two-tailed Mann-Whitney test of significance (Table 1). There is no statistically significant difference in the SCO for any UTR codon position between standard code non-ciliate alveolates and other standard code eukaryotes, using the same test of significance (Table 1). It should be noted that the use of four species of *Tetrahymena* in this study might somewhat skew the statistics representing the typical ciliate’s TSC overrepresentation scores to more closely represent the *Tetrahymena* pattern of overrepresentation. Analysis with a single composite *Tetrahymena* species (generated by averaging SCOs for each *Tetrahymena* species at each codon position), however, retains the statistically significant overrepresentation of TSCs in ciliates with reassigned stop codons for all six positions (p<0.01) against non-ciliate alveolates, and non-alveolate eukaryotes (S3 Table).

**Table 1:** Two-tailed Mann-Whitney non-parametric test comparing stop codon overrepresentation in 3’ UTRs across groups of species1.

3' UTR Position	Ciliates vs. Non-ciliate Alveolates	Ciliates vs. Non-alveolates	Ciliates vs. Non-ciliates	Non-ciliates Alveolates vs. Non-alveolates
+1	**1.66E-04**	**1.46E-06**	**1.23E-06**	0.17248987
+2	**9.54E-05**	**1.46E-06**	**1.06E-06**	0.49095878
+3	**7.17E-05**	**7.86E-07**	**5.82E-07**	0.14338112
+4	**9.54E-05**	**8.59E-07**	**6.78E-07**	0.25066688
+5	**2.84E-04**	**2.24E-06**	**2.05E-06**	0.13999068
+6	**1.26E-04**	**1.22E-06**	**9.86E-07**	0.22724746

^1^Included in this test are the SCO scores between the different groupings of 9 ciliates (with the former stop codons UAA and UAG reassigned to Gln), 12 non-ciliate alveolates (all with standard stop codons), and 60 non-alveolate eukaryotes (all with standard stop codons). The p-values were generated using the two-tailed Mann-Whitney test. As we are testing six positions for each grouping comparison we can apply the Bonferroni correction and the p-value threshold for statistical significance at the 99% level is, p<0.0016. Tests with a p-value lower that 0.0016 are statistically significant at the 99% confidence level. Significant values are indicated in bold.

### Investigation of non-UAR reassigned ciliates, and the clade *Diplomonadida*

**Fig 2:**
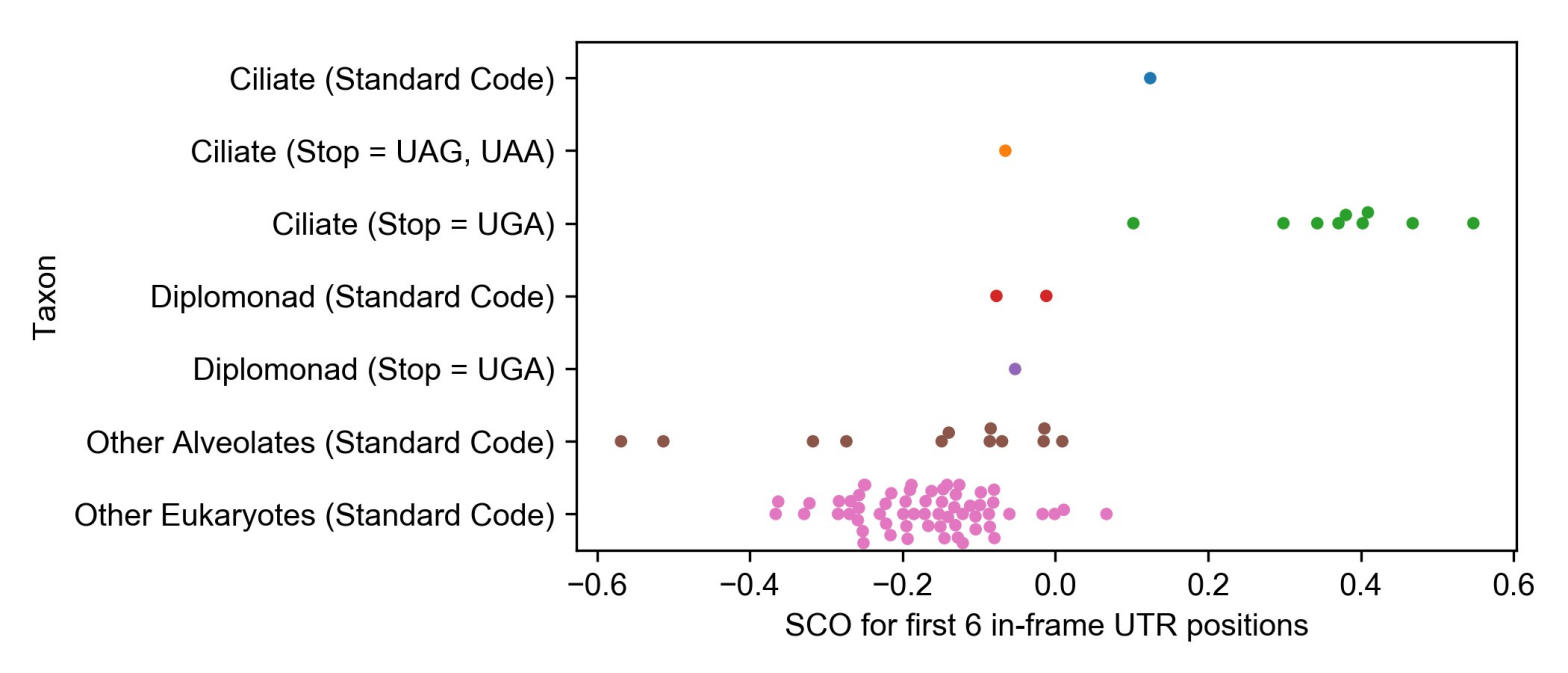
Plot of the combined SCO scores for the first six 3’ UTR positions across groups of species. Each point represents a genome, the y-axis separates the organisms based on their phylogeny and genetic code used.

In an attempt to determine whether the overrepresentation of TSCs in ciliates is related to stop codon reassignment or some other characteristic of ciliates we calculated the combined SCO score for the first six 3’ UTR positions for the standard code ciliate, *S*. *coeruleus*, and compared it with the combined SCO for other organisms (a list of all the combined SCO scores is given in S4 Table). *S*. *coeruleus’* combined SCO of 0.124 for the first six positions of its UTRs is elevated above zero and is higher than those of standard code alveolates and non-alveolates (Fig 2 and Table 2). On the other hand, its score is on the low end of UAR reassigned ciliate scores, higher only than that for *I*. *mutifilis* with a score of 0.102. *E*. *octocarinatus*, however, has a negative SCO of -0.066, lower than all other ciliate scores and lower than that of several standard code alveolates and non-alveolates (Fig 2 and Table 2).

**Table 2:** Average combined SCO scores, fraction of genes containing TSCs and fraction of genes expected to contain TSCs, across groups of species^1^.

Taxon	Average combined SCO	Average fraction of genes with TSC (%)	Average fraction of genes expected to contain TSCs (%)
Ciliate (Standard Code)	0.18	54.26	47.92
Ciliate (Stop = UAG, UAA)	-0.09	34.78	37.13
Ciliate (Stop = UGA)	0.53 (0.18)	13.03 (2.25)	8.94 (1.00)
Diplomonad (Standard Code)	-0.06 (0.07)	25.80 (2.24)	26.93 (1.09)
Diplomonad (Stop = UGA)	-0.08	9.88	10.42
Other Alveolates (Standard Code)	-0.27 (0.28)	28.00 (10.45)	32.68 (9.91)
Other Eukaryotes (Standard Code)	-0.25 (0.13)	25.25 (7.95)	29.79 (8.79)

^1^ The values in each column represent the average for the group of organisms. Standard errors are given in parenthesis following the average. Note that some groups contain a single organism, and standard deviations cannot be calculated.

The diplomonad *S*. *salmonicida* has the same reassignment of UAR to Gln seen in ciliates, so like the investigation of *S*. *coeruleus* and *E*. *octocarinatus*, the investigation of different diplomonad genomes is useful in determining the relationship between TSCs and stop codon reassignements. In our dataset, in addition to the reassigning *S*. *salmonicida*, there are two species of diplomonads that use the standard code, *Giardia intestinalis* and *Giardia muris*. The average combined SCO for the two standard code diplomonads is -0.06±0.07 and the SCO for *S*. *salmonicida* is -0.08, all lower than any UAR reassigned ciliate scores and within the range of SCOs for standard code eukaryotes.

### Comparison of TSC containing gene prevalence between organisms

**Fig 3:**
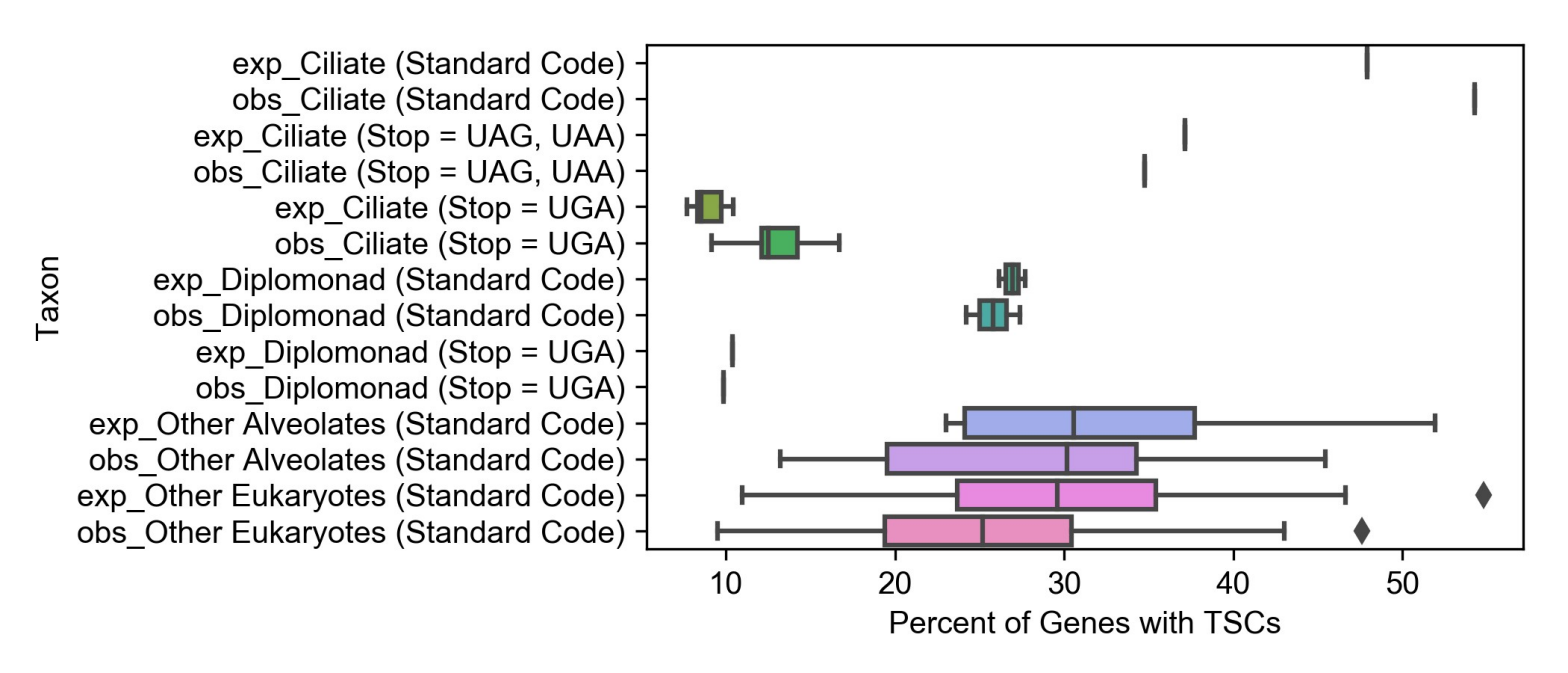
Box-plots comparing the observed and expected fraction of genes with TSCs in the first 6 positions of the 3’ UTRs across groups of species. Groups of organisms are in the y-axis, expected values have the prefix “exp_” in front of the group name and the observed values the prefix “obs”. The box plots are used to represent the combined SCO scores for the first six 3’ UTR positions for all species in each grouping, with whiskers extending to a maximum of 1.5 interquartile range, the edges of the boxes at Q1 and Q3, and the central line indicating the median combined SCO score for the grouping. Scores outside the whiskers’ range are shown as points.

Prevalence is not so informative as overrepresentation scores in determining whether TSCs are selected for in the genome, but as all TSCs should be theoretically functional, the raw percentages of genes with TSCs remain biologically relevant. Thus, we calculated the percentage of genes with stop codons in the first six positions and the expected number of genes predicted to have TSCs in the first six positions according to our null model for each organism (Fig 3, Table 2 and S4 Table).

On average, 28% of genes have TSCs in the first six positions of their 3’ UTRs for standard code non-ciliate alveolate, and 25.25% for standard code non-alveolate eukaryotes. This stands in contrast to the 13.03% in ciliates with UAR reassigned and 9.88% in the diplomonad with UAR reassigned, the difference largely due to stop codon dictionary size and AT content of the UTRs which are reflected in the expected percentages of genes with TSCs. In the UGA reassigned ciliate, 34.78% of genes bear TSCs in the first six UTR positions and the standard code ciliate, *S*. *coeruleus*, has the highest percentage of TSC-bearing genes at 54.26% (Fig 3 and Table 2).

### Prevalence of TSCs in highly expressed genes in *Tetrahymena*

Previous studies have suggested that expression level is an important factor in the presence of TSCs in yeast [20] and *T*. *thermophila* [24], although the latter study used only a limited set of genes based on the presence of mRNA in limited EST projects at the time. To estimate expression levels in *T*. *thermophila* we used a measure of codon bias, CAI, as a proxy for expression levels. Table 3 shows the number of genes with TSCs for the genes within each quartile of the CAI range.

**Table 3:** Percent of Genes with TSC in each Quartile of T. Thermophila Genes Separated by CAI Values.

Quartile of CAI Values	Total Number of Genes	Genes with TSC	Fraction of Genes with TSC (%)
1st quartile	6625	650	9.81
2nd quartile	6624	703	10.61
3rd quartile	6624	722	10.90
4th quartile	6625	971	14.66
Total	26498	3046	11.5

About 11.5% (3,046) of the 26,498 *T*. *thermophila* genes included in this analysis have at least one TSC in the first six positions following the stop codon. Table 3 shows that genes in the 1^st^ to 3^rd^ quartiles have fewer TSCs than expected from the total frequency of TSCs in the genome. The 4^th^ quartile of genes based on their CAI values has statistically significantly more genes than expected (chi-square test; null hypothesis: TSCs are as common in the 4^th^ quartile as in the whole genome; χ^2^ = 65; df = 1; p = 7.2x10^-16^).

## Discussion

There is incentive to maintain TSCs in the 3’ UTRs of genes; in the event of stop codon readthrough, TSCs prevent nonstop decay and limit the length of C-terminal extensions or eliminate the extensions entirely. In this way, the introduction of the TSCs might mitigate the deleterious effects of readthrough. Unchecked, readthrough results in the degradation of protein products or the production of proteins with C-terminal extensions that cause the protein to misfold, mislocalize, or aggregate. Relatively great selective pressures are predicted for ciliate species with reassigned stop codons which, in a few cases, have been observed to have high rates of TSCs [24].

### TSCs are overrepresented in ciliates with the UAR stop to Gln reassignment

We determined the degree of TSC overrepresentation in a number of organisms with and without stop codon reassignments. Log ratios of observed versus expected counts of stop codons, stop codon overrepresentation scores (SCOs), were calculated for the first six codon positions of organisms’ 3’ UTRs. Scores above zero indicate an excess of stop codons at that position, and potentially evolutionary selection. Overrepresentation of the UGA stop codon in initial positions following primary stop codons was observed in the first six 3’ UTR in-frame codon positions in all ciliate species studied with the UAR stop to Gln codon reassignment (Figs 1 and 2).

Not only is the overrepresentation of TSCs evident in ciliates with the UAR stop to Gln reassignment, but the trait is exaggerated in these organisms compared to standard code organisms, even other alveolates without the stop codon reassignment (Figs 1 and 2). In an analysis of 3’ UTRs from nine ciliates with the UAR stop to Gln reassignment, 12 standard code non-ciliate genomes from the same superphylum, alveolates, and 60 standard code non-alveolate eukaryotic genomes, the ciliate species are shown to have a statistical excess of TSCs in each of the first six positions of their 3’ UTRs compared to either of the other groups (Figs 1 and 2 and Table 1; p<0.001). No statistically significant difference was observed in any position between the standard code alveolates and standard code non-alveolate eukaryotes, the control comparison. Standard code species tend to have SCOs below zero for the positions analyzed. Not only does this test fail to show any discernable pattern of selection for TSCs in standard code organisms, it instead seems to demonstrate a possible bias against these codons in the 3’ UTRs for these species.

Although there is some chance that depressed standard code scores indicate active selection against 3’ UTR stop codons, it seems more likely that these scores instead demonstrate selection for other specific features over TSCs in these regions that could produce conserved secondary structures, enhance nuclear export, provide sites for miRNA binding, or enhance stop codon efficiency. It is known, for example, that the hexanucleotide sequence (taking up 33% of our six-codon window) immediately downstream of primary stop codons plays an important role in termination efficiency and certain sequences may be selected for over TSCs in those positions for many standard code organisms [5, 20].

Irrespective of depressed SCOs in standard code organisms, ciliates with UAR reassignments have high positive SCOs at the first six positions downstream of primary stop codons that are statistically greater than those generated from standard code organisms. This primary finding suggests that ciliates with the UAR stop codon reassignment have stronger selective pressures to maintain TSCs early in their 3’ UTRs than do non-ciliate standard code eukaryotes. Another evidence that these TSCs are selected for and have a biological function is that, at least in *T*. *thermophila*, they are statistically more frequent in highly expressed genes as measured by codon usage bias (p = 7.2x10^-16^).

### Possible drivers for the overrepresentation of TSCs in ciliates

#### Overrepresentation of TSCs might be related to lower chance-occurrence of TSCs in ciliates with reassigned stop codons

Because of stop codon reassignment, ciliates with only one stop codon present an interesting contradiction–they have much lower predicted and absolute frequencies of genes with TSCs, yet the overrepresentation of TSCs is far greater (p<0.001) in ciliate species with reassigned stop codons than in organisms that use the standard code. Each of the two measures of TSC representation (TSC-bearing-gene frequency and SCO) addresses a different aspect of the presence of TSCs in the genome. The former metric represents the functional reality of TSC prevalence and the fact that ciliate genomes have smaller dictionaries of stop codons and therefore are less likely to contain stop codons overall, while the latter metric eliminates the effects of UTR base composition and stop codon dictionary size to address potential evolutionary selection for the feature.

All other factors being equal, the genomes of ciliates with a single stop codon should have a relatively low likelihood of TSC occurrence due to a small stop codon dictionary (UGA vs UGA, UAA, and UAG in standard code organisms). In the data collected, only 13.03% of genes in ciliates with the UAR stop to Gln reassignment possess TSCs in the first six positions on average, compared to 28.00% in standard code alveolates and 25.25% in other standard code eukaryotes (Fig 3 and Table 2). The low likelihood of random TSC occurrence and the resulting difference in the number of TSC-containing genes might contribute to elevated 3’ UTR SCOs. For example, if a certain TSC imparts an equal fitness advantage to a standard code and alternative code organism in an identical gene and the TSC is selected for in both organisms, the relative contribution to overrepresentation will be lower in the standard code organism because the likelihood of chance TSC occurrence is higher in that genome.

Given this imbalance in chance TSC frequency, it is possible that in organisms with three stop codons, the frequency of TSCs expected by chance is enough to effectively counteract the effects of global readthrough whereas in ciliates with a single stop codon the chance frequency would not be enough, leading to greater selective pressure to maintain TSCs overall. Thus, the lower frequency of TSCs in the ciliates that do have reassigned stop codons raises the question of whether stronger forces of selection are present in species with reassigned stop codons simply to make up for the fact that fewer genes with TSCs will exist by chance.

It also must be noted that though our libraries of 3’ UTRs for the purpose of this study are standardized to the same length (20nt), ciliate UTRs tend to be much shorter than other organisms’, which also diminishes the likelihood that a stop codon will be contained within the UTRs of ciliates and could necessitate selection for TSCs early in the UTRs. On the other hand, short UTRs can improve termination efficiency as well. In the case of *C*. magnum, its short UTRs (around 20nt) seem to be essential to promote termination in ambiguous codons by way of poly(A)-eRF interactions [25].

If overrepresentation of TSCs in ciliates is indeed a consequence of stop codon reassignment and related to stop codon dictionary size, the standard code ciliate, *S*. *coeruleus*, would be expected to have relatively low overrepresentation of TSCs compared to alternative code ciliates, and *S*. *salmonicida*, a diplomonad with the UAR stop to sense reassignment, would be expected to have a high degree of TSC overrepresentation compared to standard code diplomonads. However, neither of these predictions is true: *S*. *coeruleus*, in fact, has a higher combined SCO for the first six positions of its UTRs than any other standard code organism, though its score is only higher than one ciliate with the UAR reassignment (Fig 2); and the combined SCO score for the six UTR positions for *S*. *salmonicida* is negative and well within the range of SCO scores for standard code organisms.

The high SCO score of *S*. *coeruleus* is difficult to interpret, though it does suggest that there is stronger selection for TSCs in *S*. *coeruleus* than in other standard code organisms. This runs counter to the idea that the elevated selection for TSCs seen in UAR reassigning ciliates is related to the lower number of stop codons in their genetic code and consequent lower expected frequency of TSCs: *S*. *coeruleus* has the highest predicted and observed numbers of genes with TSCs out of any organism due to its genomes’ A/T richness and use of three stop codons. This would suggest that the selection for TSCs may be a ciliate-related feature rather than a UAR reassignment related feature. On the other hand, the ciliate *E*. *octocarinatus* (UGA to Trp reassignment), shows no overrepresentation for TSCs in the first six 3’ UTR positions, which goes against the theory that elevated SCOs are a universal ciliate signature (Fig 2 and Table 2).

Thus, the addition of these genomes to the analysis is inconclusive but suggests that the overrepresentation of TSCs is not linked to some trait inherent in all ciliates given the low SCO score for *E*. *octocarinatus*, or to the UAR reassignment, given the low SCO score for *S*. *salmonicida* and the high score for *S*. *coeruleus*.

#### Selective pressures for TSCs could be related to high rates of readthrough in genes using UGA as a stop codon

Another possible force driving the selection for TSCs in ciliates with reassigned stop codons (and *S*. *coeruleus*) is a potentially higher rate of readthrough /more ubiquitous readthrough across many genes in these genomes. If there is a degree of inefficiency in the termination of translation for these ciliates, this would result in high rates of readthrough and thereby the selection for TSCs. If UGA were a less efficient termination signal than UAA and UAG, one possible reason why there may be high rates of readthrough in ciliates with UAR reassignment is their sole reliance on an inefficient stop codon, or in the case of *S*. *coeruleus*, the use of this codon at all. *E*. *octocarinatus*, the only ciliate with negative SCO score in this study does not use this codon as a termination signal.

It has been shown that the UGA codon has lower termination efficiency than UAA or UAG in *S*. *cerevisiae* and *E*. *coli* [4]. Additionally, almost every predicted candidate gene for programmed readthrough in metazoans uses UGA as a primary stop codon [32]. Recently, Eliseev et al [33] proposed that at least one Blepharisma species, *Blepharisma japonicum*, uses the standard code (while other Blepharisma species reassign UGA to Trp), and that its eRF1 can recognize all three stop codons, but that recognition is somewhat less efficient for UGA than for UAA and UAG.

Additionally, *S*. *coeruleus* has a tRNA that encodes UGA as selenocysteine (a non-standard amino acid) [34], which might make that codon potentially ambiguous in the correct context. An indication that UGA might not be an efficient termination signal in *S*. *coeruleus*, is that this organism has an extremely low usage of UGA: only about 9% of its genes use UGA as a primary stop compared to 60% for UAA or 31% for UAG (which has the same A/T composition as UGA). Perhaps this is because UAA and UAG are more efficient as primary stop codons in ciliates and have no potential to code for selenocysteine. Another indication is that the genes that use UGA as stop in this species have a slight but statistically significant higher frequency of TSCs: 57% of the genes that use UGA as stop have TSCs, compared to 54% of the genes that use UAA or UAG (chi-square test; null hypothesis: TSCs are as common in genes that end in UGA as in all the genes in the genome; χ^2^ = 11.13; df = 1; p = 0.0008).

Another factor that could reduce termination efficiency in ciliates with reassigned stop codons is lingering termination inefficiency from their putative ambiguous ancestral states, in which some subset of stop codons are read as sense in certain contexts. *C*. *magnum*’s use of all canonical stop codons as both termination signals and sense [25] is similar to the mechanism of near-cognate recognition of stop codons in readthrough. Interestingly, in the other ciliate with an ambiguous code, *Parduzcia sp*., UAA and UAG seem to have been completely reassigned, while UGA is used as both sense and as a stop codon depending on context [25]. If the ambiguous conditions represent an ancestral state as theorized [25], lingering ambiguity could explain a greater reliance on TSCs. In fact, even if there is no remaining ambiguity in ciliate codes, TSCs themselves could be artifacts from a time when the ciliates used fully or partially ambiguous codes.

On a structural level, reduced efficiency of stop codon recognition and the resulting readthrough may be related to specific changes in eRF1 domain one (the domain associated with stop codon recognition) that alter the ability of the release factor to pair with UAA and UAG codons. Studies of eRF1 indicate that the replacement of the TASNIKS and YxCxxxF motifs in the gene with equivalent motifs from alternative-code ciliates’ eRF1 sequences confer altered eRF1 specificity [35,36]. It could be that mutated residues in eRF1 and their relationship to proximal stop codon recognition motifs may play a role not only in reducing eRF1 recognition for the reassigned stop codons but also partially impairing the recognition of the UGA stop codon, thought this is speculative and does not account for the overrepresentation of TSCs in *S*. *coeruleus*. If true, these mutations could increase the rate of readthrough and consequently the selective pressures to maintain TSCs in 3’ UTRs.

Unfortunately, there are no published experimental data supporting high rates of readthrough or more pervasive global readthrough in ciliates. Regarding the efficiency of termination at UGA codons, the only study that experimentally suggests that UGA might be a less efficient termination signal in this clade was based on a study of eRF1 of a standard code ciliate, *B*. *japonicum* [33], which can use different termination codons. Additionally, ciliates that use UGA as their only termination signal should be under strong selective pressure to evolve an efficient termination at this codon.

#### Selective pressures for TSCs could be related to proteasomal regulation in the readthrough product response

Lastly, is worth mentioning that the deleterious effects of readthrough are inevitably related to the degradation of polypeptides with C-terminal extensions and, in the case of NSD, the coding mRNA that underwent readthrough. Either the proteasomal degradation of a certain protein subject to high rates of readthrough limits the function of that protein in the cell or the lack of degradation leads to potentially misfolded proteins persisting in the cell and causing more severe consequences. It is noteworthy, then, that ciliates and diplomonads have a unique set of genes devoted to proteasomal regulation. They are lacking a number of conserved genes present in almost all eukaryotes [37,38]. One of these studies cited diplomonads and ciliates as two “extreme situations”, lacking several genes that code for proteasome regulator [37]. The degradation of malformed C-terminally extended proteins in these two clades could be compromised or altered by their unique proteasome regulation which would increase the fitness benefits conferred by TSCs. However, the negative SCO scores for the ciliate *E*. *octocarinatus* and the three diplomonad genomes analyzed suggest that the overrepresentation of TSC in most ciliates likely is not due to differences in proteasomal regulation common to ciliates and diplomonads.

## Supporting information

S1 Table List of all organisms used in the study. (XLSX)

S2 Table SCO scores for the first 6 UTR positions for all organisms in this study. (XLSX)

S3 Table Two-tailed Mann-Whitney non-parametric test comparing stop codon overrepresentation in 3’ UTRs across groups of species using a single composite score for Tetrahymena species. (XLSX)

S4 Table Combined SCO scores and observed and expected number of genes containing TSCs in all genomes used in this study. (XLSX)
